# Concept of an Active Surveillance System for Q Fever in German Small Ruminants—Conflicts Between Best Practices and Feasibility

**DOI:** 10.3389/fvets.2021.623786

**Published:** 2021-02-10

**Authors:** Fenja Winter, Clara Schoneberg, Annika Wolf, Benjamin U. Bauer, T. Louise Prüfer, Silke F. Fischer, Ursula Gerdes, Martin Runge, Martin Ganter, Amely Campe

**Affiliations:** ^1^Department of Biometry, Epidemiology and Information Processing, WHO Collaborating Centre for Research and Training for Health at the Human-Animal-Environment Interface, University of Veterinary Medicine Hannover Foundation, Hanover, Germany; ^2^Clinic for Swine, Small Ruminants and Forensic Medicine, University of Veterinary Medicine Hannover Foundation, Hanover, Germany; ^3^Lower Saxony State Office for Consumer Protection and Food Safety – Food and Veterinary Institute Braunschweig/Hannover, Hanover, Germany; ^4^National Consulting Laboratory for Coxiella burnetii, State Health Office Baden-Württemberg, Stuttgart, Germany; ^5^Animal Disease Fund of Lower Saxony, Public Law Institution, Hanover, Germany

**Keywords:** surveillance system, *Coxiella burnetii*, small ruminants, one health, early warning

## Abstract

Q fever is a zoonotic disease caused by the bacterium *Coxiella burnetii*. Inhalation of contaminated dust particles or aerosols originating from animals (esp. small ruminants) is the main source of human infection. Hence, an active early warning system for Q fever in German small ruminant livestock was conceptualized to prevent human infections. First, we describe the best practice for establishing this system before evaluating its feasibility, as the combination of both evokes conflicts. Vaginal swabs from all husbandry systems with a focus on reproductive females should pooled and investigated by PCR to detect *C. burnetii*-shedding animals. Multistage risk-based sampling shall be carried out at the flock level and within-flock level. At the flock level, all flocks that are at risk to transmit the pathogen to the public must be sampled. At the within-flock level, all primi- and multiparous females after lambing must be tested in order to increase the probability of identifying a positive herd. Sampling should be performed during the main lambing period and before migration in residential areas. Furthermore, individual animals should be tested before migration or exhibition to ensure a negative status. If a flock tests positive in at least one individual sample, then flock-specific preventive measures should be implemented. This approach implies huge financial costs (sample testing, action/control measures). Hence, taking the step to develop more feasible and affordable preventive measures, e.g., vaccinating small ruminant flocks, should replace testing wherever justifiable.

## Introduction

### Infectious Agent

*Coxiella burnetii* is a small, obligate intracellular, pleomorphic Gram-negative bacterium. Because of its high tenacity, *C. burnetii* can be infectious in raw milk for 90–273 days at 4–6°C as well as in raw milk products like butter and soft cheese for 42 days at 20°C. In dust and wool, it can be infectious over 7–24 months depending on the surrounding temperature. *C. burnetii* evokes a zoonotic and mainly airborne disease called Q fever (1, 2).

### Affected Species

Humans become infected by inhalation of dust particles or aerosols contaminated with only a few *C. burnetii* organisms (3, 4). Human infections through consumption of raw milk or raw milk products like butter or soft cheese is possible but rare. There is no transmission from man to man except some rare exceptions, e.g., by blood transfusion or during childbirth (5). Infections with *C. burnetii* remain subclinical in nearly 50% of patients. However, unspecific flu-like symptoms, hepatitis, or atypical pneumonia are possible consequences of acute Q fever, which may led to hospitalization (6, 7). In addition, approximately 20% of the patients are at risk of developing chronic fatigue after an infection (Q fever fatigue syndrome, QFS) (8). In <1% of the infected patients, chronic Q fever manifests in the form of endocarditis or hepatitis with a high mortality rate. Furthermore, Q fever is a risk for pregnancy (6, 7).

In animals, many species such as wild or domestic mammals and ticks can be infected by *C. burnetii* and spread this pathogen into the environment (6, 7, 9, 10). In Germany, infected (small) ruminants kept as livestock are the main source of pathogen transmission to humans (1, 11, 12). (Small) ruminants are infected by inhalation of *C. burnetii*, while other transmission routes (e.g., sexual, intrauterine, oral, infestation with ticks) are still being studied (7, 13–16). Therefore, pathogen transmission may take place through reservoir animals, which may be part of a flock or external (e.g., wild mammals but also dogs, cats other ruminant species, or ticks). These reservoir species may have some importance in maintaining *C. burnetii* in livestock as the most important factors here are contamination of the environment by their excretes as well as direct contact with livestock on the pasture. Pathogen transmission from wildlife to humans is rarely documented but is possible and should be considered when the source of infection cannot be identified (6, 7, 10, 11, 15). Concentrating on livestock, *C. burnetii*-infected sheep excrete the bacterium mainly in high concentrations in amniotic fluid, placenta and lochia during physiological birth or abortion. Moreover, excretion in milk, feces, urine, or semen is possible (1, 13, 17, 18). Goats and cattle also excrete the pathogen at the highest concentrations in birth products or with abortion, although the excretion period is longest in milk (17, 19–22). Q fever in sheep is mostly asymptomatic with an abortion rate of approximately 5–20% (23). In contrast, Q fever in goats is connected to abortion in most cases (17, 18, 22). However, abortion in small ruminants can have several causes and is not pathognomonic for an infection with *C. burnetii*. The impact of *C. burnetii* on reproductive disorders in cattle is still under discussion (17, 19, 20).

### Pattern of Disease Occurrence

Q fever in humans and animals can be found worldwide except New Zealand and Antarctica. In Europe, an increasing trend was observed between 2012 and 2016, while from 2017 the number of confirmed Q fever cases decreased again. In 2018, 29 EU/EEA countries reported 794 confirmed Q fever cases. The three countries with the most confirmed cases in 2018 were Spain, France and Germany (24). In Germany, outbreaks of Q fever in the human population occur regularly, without nationwide spread, but often in connection with infected small ruminants. The distribution of Q fever was previously described (25–27).

In summary, the number of notified human Q fever cases in Germany has increased over the years and peaked during outbreaks since reporting obligations were regulated in 1962 (25–27). Between 2005 and 2018, the notified cases ranged between 416 and 93 each year as mentioned by the German Robert Koch Institute (RKI), without any detectable trend (24, 28). Due to the concept of passive monitoring, the number of notified cases depends on awareness by the relevant stakeholder groups and may exceed average numbers by far in the case of larger outbreaks as seen in 2003, 2005, 2008, 2010, or 2014, for example (11, 24, 25). In Germany, sporadic cases are reported nationwide, but outbreaks in the human population are reported more frequently in southern federal states and can often be linked to Q fever in small ruminants (11, 25, 26).

In comparison, the average number of notified cases in German (small) ruminant flocks fluctuated between 1970 and 2000 (26). Between 2000 and 2018, a total of 9,920 official individual cases were reported (8,359 cattle; 1,349 sheep; 212 goats) (25). Most of the notified animals were cattle, which can not only be explained by the total numbers of animals, but by the legal requirements for routine diagnostics and the different disease progression in cattle, resulting in more frequent testing of this species instead of small ruminants (26). Again, awareness of the disease is important, as illustrated by the increase of notified cases in cattle, sheep, and goats followed by outbreaks in the human population in Bad Sassendorf/Soest in 2003 and in Jena in 2005 (25, 26, 29).

Supplementary to the official reported cases, only a few surveys were conducted in the past (25). For example, the German within-flock seroprevalence was estimated at 19.3% in cattle, 8.7% in sheep, and 2.5% in goats (11). Moreover, Wolf and colleagues estimated the serological flock prevalence for small ruminants in Germany between 26 and 36.6% [*n* = 71; 14]. In comparison, in a non-representative German study, 7.8% of cattle (*n* = 21,191), 1.3% of sheep (*n* = 1,346), and 2.5% of goats (*n* = 278) tested positive for *C. burnetii* by PCR (30). Wolf et al. (14) detected that 13.9% of tested sheep and goat flocks (*n* = 71) were positive by PCR. Other studies mainly aimed at sheep farming within German federal states. Therefore, their study results vary widely due to their study design and locations (25). However, it can be concluded that detection of *C. burnetii* antibodies and the pathogen itself differs between animal species, geographic areas, and time of sampling (25).

### Zoonotic Potential

Humans are at highest risk of inhaling *C. burnetii* through distant and close contact with animals, especially small ruminants (1, 11, 12, 31). **Distant contact** may occur through geographical proximity to small ruminant flocks as well as by visits to farmers markets where small ruminants are exhibited (11, 29, 31). As Q fever is an airborne disease, the pathogen can be spread over longer distances by wind and may pose a risk for human infection (11, 31). Therefore, the greatest risk of infection is within a radius of 2–4 km from the source of the pathogen. Moreover, in gale force winds, *C. burnetii* may reach distances up to 18 km (31). Migration of small ruminants through residential areas was identified as a potential risk factor (32). Therefore, it may be a challenge to identify the infection source in human cases that did not have any obvious or known contact with sheep or goats. A higher risk of transmission from small ruminants to humans can be observed in spring/early summer due to the out-of-season lambing of some sheep breeds on pasture (11, 31, 33). Furthermore, **close contact** with contaminated products such as afterbirth or contaminated wool during sheep shearing may also be sources for human infections that can occur sporadically, e.g., animal owners, their family members, or employees (33, 34). Moreover, employees of slaughterhouses or laboratories can become infected at the workplace. Infections by consumption of contaminated raw milk (products) is possible but rare (5). Also possible, but even rarer, is infection through live cell therapy (35).

### Rules and Regulations

On an international level, the World Organization for Animal Health (OIE) lists Q fever in the category of multiple species diseases (36). Moreover, OIE recommends protocols for diagnostic testing and vaccination of Q fever in small ruminants to prevent human infections (5). On the level of the European Union, data sampling of zoonoses in humans is regulated under Decision No 211/98/EC and coordinated by the European Centre for Disease Prevention and Control (ECDC). Moreover, Q fever is included in the Community Summary Reports on zoonoses since 2005. To harmonize information about Q fever within the European Union, a scheme for the monitoring and reporting of Q fever in animals was developed in 2010 under Directive 2003/99/EG. This report was prepared by the European Food Safety Authority (EFSA) in collaboration with the ECDC and EFSA's Zoonoses Collaborating Centre (32).

On the national level, Q fever in humans is a notifiable disease with inception of the German Protection against Infection Act (IfSG) in 2001 (25, 37). In accordance with the German National Animal Health Act (TierGesG) and the German Regulation of Notifiable Animal Diseases (TKrMeldpflV), only the direct detection (by culture or PCR) of *C. burnetii* in small ruminants and cattle has to be reported to the local veterinary health authority (25, 38, 39). All notifications are reported in the Animal Disease Reporting System (TSN) by the local veterinary health authorities and contain the date of detection, the species, the flock and its owner as well as the county/city concerned (25). Apart from official rules and regulations, the RKI published recommendations for Q fever after a huge sheep-associated outbreak in 2006 (9, 29). Moreover, an interdisciplinary research association in the German federal state Baden-Wuerttemberg published recommendations for the management of Q fever cases and outbreaks (40). Furthermore, the German Federal Institute for Risk Assessment (BfR) recommends pasteurization of raw milk before consumption and food processing if a *C. burnetii* infection is diagnosed within a ruminant flock (2). A recommendation published by the German Ministry of Food and Agriculture (BMEL) is available that involves hygiene measures in the case of Q fever cases in (small) ruminants (41). However, these recommendations are not mandatory and cannot be imposed by the local veterinary health authorities. In comparison, the local public health authorities may impose regulatory measures for animals based on the IfSG if humans are infected.

### Rationale for Using a Monitoring and Surveillance Systems

The German legislation underlines the rationale for using a monitoring and surveillance system (MOSS) for Q fever as it defines this zoonosis as a notifiable disease in both (small) ruminants and humans (37–39). Hence, both MOSS's that are currently in place can be defined as passive monitoring systems, which focus on human health as the predominant rationale instead of health or production effects on animals (25, 42). However, the passive monitoring system of Q fever in small ruminants does not serve as reliable protection for humans against infection. This is especially because infection in small ruminants may proceed with unspecific symptoms or even asymptomatically (17, 18). As a passive reporting system does not detect these kinds of diseases very well, we assume that the occurrence of Q fever in small ruminants is heavily underestimated in Germany (43, 44). To prevent new infections in humans, it is important to gather reliable information about the current Q fever status of small ruminant flocks. With this knowledge, flock-specific action and control measures can be induced, while these measures effectively prevent new cases in the human population, as transmission risk will be reduced (5, 38, 39). It could be discussed that the low number of notified cases in the human population does not directly impose the need for action. However, underreporting of Q fever in humans has to be assumed, as symptoms are unspecific, and zoonotic infections are not high on the list of differential diagnoses (30, 45). Due to the obvious risk for human infection, there is a need to improve passive monitoring in small ruminants toward active surveillance, including measures for the control of Q fever (5, 42, 44). Therefore, the objective of this paper was to conceptualize such a MOSS as an early warning system for Q fever in small ruminant livestock in Germany and to evaluate the concept with regard to feasibility.

## Methods

To comply with international standards, previously developed checklists for the design of a MOSS in the animal health sector were used and modified (42, 43, 46–48). Referring to these international standards, we summarized the main characteristics and the chosen options of our concept in Tables 1, 2. First, we started by defining the **hypothesis and aim** of our MOSS. Afterwards, we discussed the **type of system** in detail. All further options in terms of the described characteristics of this concept are contingent on this aim with regard to international literature about *C. burnetii*, national legislation and regional differences in husbandry of small ruminants. Next, we focused on the characteristics of the **target population** for this MOSS, which is split into the topics ***animal species***, ***sex and age***, ***region*, **and ***husbandry***. After this, we determined the **dependent variables** including the topics ***disease stage***, ***unit of interest*, **and ***diagnosis***. Then, the **independent variables and confounder** were discussed. In the next step, we focused on the **sampling technique and sample size** at the ***flock*** ***level***and ***within-flock level***as well as ***sampling time***and ***bias***. As Q fever bears a serious risk for the human population, we discussed an **action and control plan** ***in*** ***case of a positive***or ***negative flock status***. As **implementation and evaluation** of a MOSS are important issues at the time of concept planning, we discussed the involvement of ***stakeholders***as well as ***economic considerations***in the context of this concept.

**Table 1:** Concept of an active surveillance system for Q fever in German small ruminants—conflicts between best practices and feasibility.

**Characteristics (Topics)**	**Chosen options**
Hypothesis	Small ruminants are the main source for Q fever infections/epidemics in the human population in Germany
Aim	Early warning system for Q fever in small ruminant flocks to prevent Q fever infections in the human population
Type of system	(Voluntary) active surveillance system in small ruminants with individually adapted action and control plan
Target population	All female reproductive small ruminants within the flocks regardless of the husbandry systems in Germany
Dependent variable	The flock status of *C. burnetii* shedding detected by PCR of pooled vaginal swabs in female small ruminants in Germany—with the flock-status defined as positive by at least one pool-sample testing positive
Independent variables	Factors influencing the flock status of *C. burnetii* shedding: - Preliminary information (suspicious symptoms, antibody activity, vaccination) - Target population (sex and age)
Sampling technique and sample size	Multistage risked-based sampling
At the flock level	Testing all flocks at risk to transmit the pathogen to public (reproduction, close contact, distant contact)
At the within-flock level	Testing all primi- and multiparous females after lambing in order to increase the probability of identifying a positive herd
Sampling time	During the main lambing period and before migrating in residential areas
Bias	- Selection bias - Information bias
Action and control plan	(Voluntary) individually adapted control plan in positive tested flocks
In the case of positive flock status	- Prevention of close contact between flocks and the public - Prevention of distant contact between flocks and the public - Hygienic measures on the farm - Flock-vaccination - Continuous testing - Examination of the people living and working on the farm
In the case of negative flock status	Continuous testing

**Table 2:** Implementation and evaluation of an active surveillance system for Q fever in German small ruminants—conflicts between best practices and feasibility.

**Characteristics (Topics)**	**Chosen options**
Stakeholders	Small ruminant sector - Animal owners, their employees, family members - Regional/national/breeding associations - Animal traders, abattoirs, dairies, and sheepshearers Veterinary health professionals - Veterinary health authority - Veterinary practitioners/animal health services - Laboratorians Human health professionals - Human health authority - Physicians - Laboratorians Policy makers Animal Disease Funds Public
Economic considerations	Financial costs - MOSS coordination - Sample collection - Sample testing - Costs from modifying usual flock management and housing practices - Vaccination costs - Loss of income Financial benefits - Saved costs for physicians, laboratories, medicine, hospitalization - Saved non-productive time of humans - Quality awareness - Saved cost for action measures Emotional costs - Stigmatism of people in the small ruminants sector - Existential fear of people in the small ruminant sector - Panic in the public Emotional benefits - Knowledge about flock status and necessary consequences - Responsibility of animal owners toward their fellow humans - Gain in confidence of the public in the animal owners - Zoonosis prevention/One Health

The referred literature was assessed in a non-systematic search strategy using the search database Web of Science (http://apps.webofknowledge.com) performed in English. Investigated search terms were defined and combined with the Boolean Operators AND and OR.

To report about our concept of an active surveillance system for Q fever in small ruminants, the STROBE Statement was used as a guideline (49).

## Concept of the Moss

In this paper, we compile a concept of an early warning system for Q fever in small ruminants in Germany. First, we describe the best practice for this MOSS before taking the next step to address feasibility, as the combination of both evokes conflicts. The basic points of the MOSS are summarized in Tables 1, 2.

### Hypothesis and Aim

To conceptualize a MOSS, the hypothesis and the aim are mandatory to define first (42, 43). These generate the basis for every subsequent decision regarding the design of the concept. Q fever has a non-specific aetiopathology as well as little effect on the health status of small ruminants. Nevertheless, there is no active MOSS for Q fever in small ruminants, which is mandatory in all federal states. Based on these facts, we assume that the health status of most small ruminants and thus the potential sources of human Q fever infections in Germany are unknown. As only a few *C. burnetii* organisms can initiate active infection in humans (3), it is imperative to detect a possible source of transmission as soon as possible. Therefore, “early detection in a defined animal population” is the predominant purpose of the MOSS presented here (5, 36, 46).

### Type of System

MOSSs are used to gather health data of a defined population (47, 48). However, it should be discussed whether “passive” or “active” data collection is desired and if action should follow a positive finding, i.e., “monitoring” or “surveillance.”

In the case of our MOSS, “monitoring” would result in the observation of the Q fever status in small ruminants without any control activities in the case of positive findings (42, 43, 47). However, as we are concerned with a zoonotic agent with considerable human health impact, monitoring without control activities would foil the principles of risk prevention and veterinary public health. Hence, “surveillance” system is preferred here. Those control activities can be either mandatory or voluntary. For mandatory implementation of control activities, rules, and regulations are necessary. In the case of human outbreaks caused by small ruminants or cattle, veterinarians and human local health offices have to work closely together using the IfSG. However, German law does not regulate consistent control activities in the case of Q fever in small ruminants without a link to human Q fever cases (37–39). A competent public health authority can take necessary measures to avert imminent danger only if facts are established, which might lead to the occurrence of Q fever in humans, or if it can be assumed that such facts exist (37). Hence, regulations need modifications. In the meantime, surveillance is contingent upon the voluntary implementation of control activities by animal owners. However, a surveillance system with voluntary control activities calls on the responsibility of animal owners for people in their environment. For the success of this MOSS concept, further considerations presume a 100% participation rate.

Furthermore, it is important to decide whether a “passive” or “active” data collection must take place for this MOSS (42, 43, 47). In the case of a “passive” MOSS, a reporting cascade is necessary to gather health data (43). Such a “passive” method would be an inexpensive way to get information about the Q fever status of small ruminants in Germany because the veterinary health authorities would not need to plan data acquisition, to take diagnostic tests or finance the work of sampling and laboratories. However, this “passive” system contains many sources of error as it is based on the idea that animal owners and practicing veterinarians recognize clinical signs of Q fever very easily and screen the population of interest. Therefore, passive monitoring would overlook small ruminants with positive Q fever status. Furthermore, different knowledge about Q fever and different motivations to report positive flock status negatively influence the quality of health data (42, 43, 50). Animal owners may be less motivated if they distrust the system, especially if consequences after reporting a positive Q fever status are unforeseeable [e.g., stigmatzation; (43)]. Therefore, the reporting cascade would not work reliably for this disease. In contrast, an “active” MOSS collects health data in a systematic way or by regular recording (42). For the active collection method, it is typical to define a specific aim of the MOSS, to use a formal sampling process in a specific population and a specific time of collection as well as standard tests to detect positive findings (42, 43). In general, the local veterinary health authorities are responsible for the coordination of this regular and periodic collection of health data (43). It is important to invest both money and time in structured data acquisition to get valid information about Q fever status in a favorable cost-benefit ratio (46–48). Hence, this leads to an “active” system of data collection in the case of our MOSS. An active MOSS is not restricted to clinical cases with typical symptoms, which is an important advantage in the case of Q fever in small ruminants. Another positive effect for the quality of the data is the control of sampling scheme and information collection by a competent veterinary health authority that guarantees statistically based objectivity as well as increasing comparability of results (42, 43). In conclusion, this MOSS should be implemented as an active surveillance system.

### Target Population

The target population is defined as the population that is the focus of a study (42, 51). For example, this can include the whole population at risk that is susceptible to a specifical disease or just a subgroup with special qualities of this population at risk (42). If the target population is not clearly defined, individual interpretation of study results can lead to bias and differential perception of the results (51). To define the target population of this MOSS, we divided their characteristics into the topics ***animal species***, ***sex and age***, ***region*, **and ***husbandry*** (46–48).

#### Animal Species

Although various domestic and wild mammals can be reservoirs for *C. burnetii*, the focus of this approach is on small ruminant livestock (6, 7, 9, 10). On an international level, (aborting) dairy goats have been the reason for the biggest Q fever outbreaks in humans. However, in Germany most small-scale epidemics have been caused by nondairy sheep (25). Because sheep and goats are often kept together (especially in southern Germany) and because the number of goats is increasing across the country (14, 25), this MOSS has to focus on both species. The German Federal Statistical Office reported 19,556 farms with 1,834,275 sheep and 9,808 farms with 138,810 goats in 2016. Unfortunately, no official number about these farms keeping sheep and goats together is available (52, 53).

#### Sex and Age

Possible differences regarding the transmission risk to humans between sex and age groups have to be considered when defining the target population. Both female and male small ruminants can be infected by *C. burnetii* and shed the pathogen via different pathways (13, 14, 18, 21, 22, 54). However, the highest pathogen concentrations are shed in amniotic fluid, placenta, and lochia during physiological birth or abortion and in the milk of small ruminants (18, 21, 22, 54). Hence, female small ruminants after lambing are the subgroup with the highest risk for humans to acquire infection and therefore are focused on in this MOSS. In Germany, approximately 64.42% (*n* = 1,181,560) of the sheep population and 63.72% (*n* = 88,451) of the goat population are reproductive females (52).

#### Region

As the pattern of disease occurrence of Q fever in humans and small ruminants in Germany was previously described (25–27), the reported cases and studies show that Q fever is endemic in Germany (11, 25–27). Therefore, the MOSS should be implemented nationwide. In Germany, rules and regulations for the implementation of a MOSS are split into national, federal, and district tasks. In the districts, the lowest administrative level, veterinary and public health authorities are responsible for general coordination and implementation of disease prevention in animals as well as in humans. These veterinary and public health authorities on the district level can rely on (communication) structures between different stakeholders, which are needed for the implementation of this MOSS (see chapter “Implementation and evaluation”). As the wind and the distance between settlements and small ruminants have an impact on Q fever transmission (11, 31), this MOSS mentions urbanization, density of livestock, and individual weather conditions in different regions of Germany and appeals to the cooperation between neighboring districts in the case of positive findings. In conclusion, this MOSS must be implemented nationwide with the administrative districts as the lowest subregional structure being responsible for implementation of action and control activities.

#### Husbandry

The current situation of sheep and goat husbandry in Germany was previously described by Bauer et al. (25). In short, the distribution of animals, farms and breeds differs between German federal states (25). Moreover, most German small ruminant farms practice a combination of management systems including grazing enclosure, milk production, and shepherded and migrating flocks, which makes husbandry very inhomogeneous (50). In addition, small ruminant husbandry is mostly associated with **reproduction** for lamb production as well as with landscape conservation and protection. Both are connected with **distant contact** between small ruminants and the public due to lambing and grazing on pasture and the proximity to residential areas. Migrating flocks, which change their location regularly or even cross administrative district borders, are particularly noteworthy here. Furthermore, **close contact** between small ruminants and the public takes place in petting zoos, family farm vacations, and animal-assisted education or therapy. These risk factors for public health will be discussed in the “sampling technique and sample size” section of this manuscript. Finally, this MOSS needs to focus on the whole range of small ruminant husbandry, as there is an overall risk of transmission to humans.

### Dependent Variable

Conceptualized as an early warning system, the dependent variable for this MOSS is the status of Q fever in small ruminants.

#### Disease Stage

To describe a disease status in general, a MOSS can focus on different disease stages such as the infection, the occurrence of symptoms, the presence of antibodies, or the excretion of the pathogen itself. As we have to consider the zoonotic potential of Q fever, pathogen shedding by small ruminants must be identified as early as possible. Since clinical symptoms or the presence of antibodies cannot be connected to the occurrence of pathogen shedding (12, 18, 21, 32, 44), this MOSS has to focus on the detection of pathogen shedding itself. As mentioned in the topic **sex and age**, the perinatal period is the time of highest risk for human infections (18, 21, 22), and this MOSS focuses on the detection of current pathogen shedding early after lambing.

#### Unit of Interest

“The individual (animal), pen, herd, flock, or farm” or another defined structure may be chosen as an unit of interest (42). Choosing the individual animal as the smallest unit of interest is useful to detect sporadic diseases. Therefore, this strategy is valuable for a disease that is not very contagious. However, Q fever is a contagious disease and can be spread easily within flocks. This means that the occurrence of more than one *C. burnetii* shedding individual within a group of animals is very probable. Choosing the status of pathogen shedding at the flock level, the probability of a positive flock status may change over time with the composition of individuals within the flock (51). As farms may manage more than one flock, and flocks may be housed and handled differently, flocks should be the most appropriate unit of interest here (5, 51, 55). Due to the high concentration and prolonged time of *C. burnetii* shedding, a single *C. burnetii*-infected individual may be a sufficient risk for human infections (3, 29). Therefore, a single animal testing positive is sufficient to designate the flock as positive for *C. burnetii* shedding (14, 44, 55). Conclusively, the unit of interest is the flock status of pathogen shedding defined by at least one individual-sample testing positive.

#### Diagnosis

Different diagnostic tests are available for direct detection of *C. burnetii*. Only specialized labs perform bacterial cultivation. However, PCR is the most feasible diagnostic test for this MOSS because primer and probe sequences as well as ready-to-use kits are commercially available, and results may be provided with high speed and accuracy (i.e., high specificity and sensitivity) (1, 5, 25). PCR has been used to detect *C. burnetii-*genome fragments in feces, however further research is necessary to confirm feces as adequate test material to detect acute pathogen shedding in small ruminants (56, 57). As an officially approved diagnostic test, PCR is able to detect *C. burnetii-*genome fragments in milk samples, organs of fetuses, birth products, vaginal swabs, and ticks (25, 58). However, milk production is present on only 2.68% (*n* = 524) of all sheep farms and comprises only 0.98% (*n* = 17,999) of the sheep population in Germany in 2016, making this sample material unsuitable for this MOSS (52). In addition to sampling of fetuses and birth products, sampling of vaginal swabs means additional effort and cost, but vaginal swabs provide a high-quality sampling material (5, 25, 59). Furthermore, a vaginal swab can be assigned to an individual animal, so that subsequent measures can be implemented individually (e.g., follow-up testing). In addition, pooled samples of vaginal swabs are applicable to reduce temporal expenditure and financial costs (42). Conclusively, diagnosis must be carried out by conducting pooled vaginal swabs testing by PCR.

### Independent Variables and Confounder

Factors influencing the probability of a flock testing positive for pathogen shedding were already discussed before and are only listed here for reasons of clarity:

- Preliminary information: Flocks already known to show **suspicious symptoms such as abortion** at the flock level [the abortion rate ranges between 5 and 90% of pregnant females, while 5–20% is common in sheep flocks, and high abortion rates occur only in some goat flocks (23)] may be an indicator of current and ongoing infection and shedding. Moreover, previously known **antibody titers** in animals may be an indicator of current or ongoing infection and shedding in a flock. This may increase the probability of a flock testing positive for current shedding but there is no guarantee (1, 59). Although previous **vaccination** heavily influences the antibody activity, it also reduces the amount of pathogens shed (5).
- Target population: Female animals with perinatal status are most likely to shed high amounts of the pathogen (see chapter “Sex and age”) (18, 21).

### Sampling Technique and Sample Size

First, it has to be decided which procedure is applicable for the aim of this MOSS: a census or a sample. The main disadvantage of a census is its great cost in terms of time and money; conversely, an advantage of sampling is its low cost, while leading to reliable estimation of the target population if the sample is selected correctly (42, 51). A solution is to conduct a risk-based sample. This enables lower costs and monitoring of the most relevant samples. Due to the well-known cluster effect of (n) animals in (m) farms, multistage sampling has to be considered, where first the number of flocks and then the number of animals per flock are determined (51).

#### At the Flock Level

Concerning risked-based sampling, flocks with the following characteristics should be investigated predominantly:

- **Reproduction** in general, but especially when lambing will happen on pasture: Information about the flock management (lambing location i.e., lambing in stable vs. on pasture) as well as the time of parturition (estrus synchronization/artificial insemination, lambing season) must be queried (33).
- **Close contact** in general, but especially when female and pregnant small ruminants are to be exhibited: Information about exhibitions (i.e., conformation shows, farm vacations, open house days). Furthermore, all reproducing female small ruminants kept for petting zoos, animal-assisted education and therapy must be identified and investigated in particular (29).
- **Distant contact** in general, but especially when migration is performed in residential areas: Information about the migration of flocks must be acquired, and the migrating flocks must be tested regularly (31, 33).

As it can be assumed that these characteristics concern most of the German small ruminant flocks, this risked-based sampling may almost be a census.

#### At the Within-Flock Level

Concerning risked-based sampling, all females after lambing should be investigated predominantly. Assuming that the flock has a positive status given that one individual is shedding *C. burnetii*, we calculated the required sample size (n) to identify freedom from disease considering the absolute number of primi-/multiparous females per flock (N) [see Supplementary Material; (5, 36, 51)]. Our calculation shows that the difference between census and sample (N-n) is minimal and therefore this risked-based sampling is almost a census (see Supplementary Material).

#### Sampling Time

Flock level testing should be done during the main lambing period and before migrating into residential areas at least once a year. In addition, an ultrasound examination should be carried out before individual animals are exhibited or transported/slaughtered. Animals in the last trimester of pregnancy should not be exhibited to prevent spontaneous abortions or births near humans and should not be transported/slaughtered to prevent infection of animal traders and abattoirs.

#### Bias

A sampling process always leads to an uncertainty about the gathered information because a conclusive statement in response to a research question is only available for the sampled individuals (42). To assess the quality of this MOSS, this section discusses possible sources for bias.

**Selection bias** describes the difference between an estimation and the truth (60). In case of this MOSS, the selection bias may originate from the sampling technique described above. First, it is important to mention that this MOSS currently depends on voluntary participation of the different stakeholders. Poor participation by these stakeholders could introduce selection bias such that only interested animal owners participate. This could bias the percentage of flocks testing positive toward an overestimation, if these persons already know about their flock having a problem with Q fever. However, it could also bias toward an underestimation, if these persons focus their flock management on a perfect hygiene strategy and therefore participate to prevent further *C. burnetii* infections. A percentage over- or underestimation would not impede the aim of this MOSS, which is to identify as many herds as possible in total that shed *C. burnetii*. Furthermore, if the system becomes obligatory, this bias will be settled. Next, veterinary health officers have to administer the risk-based principle of flock selection and individuals within the flocks. For example, sampling of non-mated female small ruminants, which do not lamb, or sampling of mated female small ruminants during pregnancy and late after lambing also may lead to underestimation. Although this selection process can be supported by the use of a questionnaire, it depends on subjective decisions and may be a source of possible bias. Beyond this, correct assignment of high-risk flocks depends on animal owners' provision of correct information.

**Information bias** describes the difference between the estimation and the truth that originates from over- or underestimation of results (60). In the case of this MOSS, the greatest information bias is because a few animal owners do not know the time of lambing, as fertile male small ruminants are constantly in the herd. Therefore, the information about time of lambing can only be communicated to the veterinary health officers when it has already taken place. Very good communication between animal owners and veterinarians is therefore a prerequisite for obtaining samples in a timely manner. Further information bias may originate from error classification of pools resulting in false classification of the flock status. This depends on the sensitivity and specificity of the chosen diagnostic test (60), which are very high in the case of PCR testing (1, 5, 25). As *C. burnetii* shedding occurs sporadically, test results can miss a positive individual and therefore the positive status of a flock. By planning to test herds completely and during a time when identification of positive animals is most likely, single false-positive or negative test results can only modify the estimated disease status on the flock level if shedding occurs in very few animals or if the flock is small. Therefore, as the test accuracy is comparably high and pools instead of individual animals are investigated, the test performance does not have to be considered as relevant bias for the determination of disease status.

### Action and Control Plan

Recommendations for action and control measures regarding Q fever in small ruminants have been published elsewhere (1, 5, 12, 25, 33, 40, 41). However, German flock management and husbandry vary considerably between each small ruminant (production) system, resulting in a varying risk of pathogen transmission to the human population (5). Therefore, individually adapted action and control plans are preferable over general recommendations and should be developed with the cooperation of veterinary and human health professionals and the animal owners.

#### In the Case of Positive Flock Status (Pathogen Shedding in a Flock)

If *C. burnetii* shedding is detected, immediate and long-term actions must be defined in a flock-specific action and control plan (41).

Most importantly, prevention of close contact between the positive flock and the public means that animal **exhibitions** as well as **unauthorized persons in the flock** (i.e., farm visits, vacations, open house days) have to cease until the flock is proven negative again. **Authorized persons working in the flock** (animal owners, their employees, animal traders, abattoirs, dairies, sheepshearers) must protect themselves with **personal protection equipment;** wearing FFP3 respirators is most important, and protective work clothing should only be worn within the specific flock (12, 33, 40, 41).

Similarly important is the prevention of distant contact between the flock and the public. Therefore, the animal owners have to organize **lambing inside a stable** and to **store contagious materials** such as afterbirth or aborted material in safe containers until rendering. In addition, **cleaning and disinfection** of lambing areas and stables are necessary to prevent pathogen dissemination. Furthermore, **dung and litter** have to be covered for 9 months before spreading it on farmland (12, 33, 40, 41). Although the alimentary infection pathway is unlikely (2), selling and consumption of **raw milk and raw milk products** must be prohibited (12, 40, 41). Small ruminants must not be used as a source for **live cell therapy** (35). No **migrating** should be allowed, and the flock should be **kept as far as possible from human habitation** until the flock is proven negative again (31). **Shearing and storage of wool** have to take place in a closed room while wearing personal protection equipment. Contaminated wool must be destroyed in a rendering plant (31, 33, 40, 41).

Moreover, an action that concerns the individual animals itself is **vaccination**. However, phase I vaccine should be preferred over phase II vaccine as it is more effective (61). Although phase I vaccination cannot stop the shedding of *C. burnetii*, it can reduce pathogen shedding (1, 17, 33, 61). Oxytetracycline treatment is not recommended in the case of Q fever in small ruminants, as it does not stop pathogen shedding (25, 61).

As Q fever occurs sporadically and may reoccur after some time, a flock with a positive status should be **retested** for at least the next two lambing seasons (22). To prevent further unnoticed pathogen spread, the possible source of infection should be investigated [**tracing on** and **tracing back**, **wildlife as pathogen reservoir**; (10, 12, 33, 34, 45, 54, 61)].

#### In the Case of Negative Flock Status

If the status of a flock is negative, *C. burnetii* shedding was not detected by PCR of vaginal swabs and transmission to the public is currently unlikely. However, as Q fever occurs sporadically, **annually recurrent testing** is necessary. In cases of **increased risk for transmission** to the public, additional testing should be applied. Furthermore, **animals in their last trimester of pregnancy must not be transported**, e.g., for exhibition or slaughter, in order to avoid pathogen contamination by spontaneous lambing or slaughter. Accordingly, an ultrasonography examination to determine the pregnancy status of an individual animal has to be carried out prior to transportation.

### Implementation and Evaluation

To implement and evaluate this MOSS, it is important to discuss which ***stakeholders***are potentially concerned and which ***economic considerations***should be regarded.

#### Stakeholders

Stakeholders who are affected in some way by this MOSS are different subgroups working in the small ruminant sector as well as veterinary and human health professionals, policy makers, Animal Disease Funds and the public itself (5, 29, 45, 55, 62–64).

The small ruminant sector first includes **sheep and goat owners, their employees, and family members**. Small ruminant owners are organized within **regional and national (breeding) associations**. These associations represent the opinions of their members and are important counterparts to get in contact with small ruminant owners and amplify information regarding MOSS implementation. As long as participation is voluntary, animal owners have to actively agree to take part. Participation will be influenced by the good communication between different stakeholder groups and awareness of the importance of this MOSS for public health. Therefore, we discussed the idea of closely linking voluntary Q fever monitoring to another already well-established (and mandatory) monitoring program for brucellosis with a group of representatives from small ruminant owner associations. It became clear that awareness about the impact of this disease for public health currently is not high enough to trigger willingness to participate. The representatives emphasized that small ruminant owners are already overloaded with regulations and legal documentations and cannot justify further workload and restrictions, which are not predictable in the case of a positive flock status. Hence, it has to be concluded that prior to and in parallel with implementation of this MOSS, great effort must be put into raising awareness, dismantling barriers and fears and increasing knowledge of Q fever and exactly how the MOSS will work. Furthermore, possible economic benefits of a negative test result need to be discussed and emphasized to create an incentive for participation (see chapter “Economic considerations”).

Further groups in the small ruminant sector that have to be considered are **animal traders, abattoirs, dairies, and sheepshearers**. These groups need to know the status of flocks in order to adapt their working habits when handling positive flocks with regard to personal protection equipment (i.e., wearing FFP-3 breathing masks) or when processing material derived from small ruminants [i.e., pasteurization of raw milk, separate slaughter, handling and selling of wool; (33, 40, 41)]. Some professionals might even have to postpone or cancel their work in small ruminant flocks that tests positive (e.g., sheepshearers) due to self-protection.

Veterinary health professionals includes the **veterinary health authority officers** who have to organize steps such as selection of most relevant flocks as well as the documentation and analysis of test results. Elaboration of flock-specific action and control plans is another of their tasks. However, these steps must take place in cooperation with the animal owners. At this point, **veterinary practitioners** function as a link between the veterinary health authority and animal owners, as they know the animal owners as their customers and therefore can advise which measures have to be implemented for the affected flock. In addition, veterinary practitioners must perform the vaginal swab sampling on behalf of the veterinary health authority. Since an animal health service exists in most German federal states, these tasks can be transferred to the practicing veterinarians of this organizational unit. **Laboratories** are necessary to do the diagnostic testing of these swabs. To implement necessary safety measures at the laboratory, samples have to be packed safely and accompanied by meaningful preliminary reports.

As Q fever is a zoonosis, human health professionals are additional stakeholders in this MOSS. Therefore, the **public health authority officers** and their colleagues at the veterinary health authority must cooperate. In the case of a positive flock status, an exchange of information must take place automatically, as this provides an early warning to draw attention to possible Q fever cases in the human population. Hence, the public health authority officers should forward information to **physicians** to raise awareness and alertness, without stigmatizing the animal owners and their families. Furthermore, **laboratories** in the human health sector are involved if human cases occur, and they must be informed to be alert about the zoonotic potential as well.

Next, policy makers are an important group of stakeholders in this MOSS. Due to recent German legislation, Q fever is only monitored via a passive MOSS. Hence, implementation of any new surveillance attempts depends on the voluntary participation of animal owners and veterinary health professionals. Therefore, legislation needs to be revised, and an active MOSS has to become mandatory in order to protect the public effectively.

Furthermore, the revision has to include subsidies for animal owners in cases of positive herd status. This leads to the Animal Disease Funds as further stakeholders. These public-law institutions are regulated nationwide by the TierGesG, but within the federal states the reimbursement of costs for monitoring and combating animal diseases varies (38). Therefore, federal legislation is necessary to regulate the subsidization of animal owners by these institutions in the case of positive flock status.

Finally, the public has to be mentioned as a stakeholder group. If there is a risk of a Q fever outbreak in the population, the population should be informed about possible risk factors and preventive behavior. Therefore, it is important that the public relations department of each district cooperates with the local press to inform the public without generating panic or causing stigmatization of small ruminant owners (62, 63).

#### Economic Considerations

Decisions about this concept are driven by economic considerations that affect all different stakeholder groups.

Financial costs evolve from MOSS coordination, sample collection and testing as well as action and control measures (see Supplementary Material). Therefore, the conflict between best practice and feasibility is notable.

For **MOSS coordination**, labor costs for veterinary health authority employees have to be assessed prior to implementation. Here, the effort to collect basic information about the risk-status of the flocks within each governmental district is considered the most time-consuming task. Calculating the financial costs of **sample collection, sample testing**, and vaccination, as an **action and control** measure, we considered the German veterinary fee regulation (GOT), the fee regulation for administration/consumer protection and veterinary health authority (GOVV) in Lower Saxony and the German permanent vaccination commission for veterinary medicine (STIKO Vet) [see Supplementary Material; (65–67)]. As **sample collection** has to be carried out by veterinary practitioners on behalf of the veterinary health authority, labor costs, driving costs, materials (vaginal swabs) and sample shipment must be taken into account. Additionally, costs for **sample testing** by the laboratories must be considered, whereby pooling of samples saves costs (see Supplementary Material). Concerning **action and control**, all implemented measures have to be supervised by the veterinary authorities, which causes additional personnel costs. For **vaccination**, we assumed four euros per 2 ml dose for a small ruminant individual (25). Further, we assumed that only the gimmers, replacement animals, and purchases of the flock (replacement rate of 20%) need an initial immunization. Here, two doses at intervals of 3 weeks were calculated. If possible, vaccination has to be completed 4 weeks before mating (61), while the other 80% of the flock gets only one booster vaccination per year. **Sample collection, testing, and vaccination** of all reproductive females (*n* = 1,270,011) in the German small ruminant population include 26,090,430 euros per year in this calculation [see Supplementary Material; (52, 53)]. Moreover, animal owners would face (additional) **costs from modifying their usual flock management and housing practices** in order to ensure that pathogen transmission will be prevented. Rendering of contaminated materials may cause additional costs. Furthermore, and most importantly, the **loss of income** for the affected animal owners has to be considered before implementing this MOSS, as well. Financial damage may be substantial depending on the purpose of use and the market value of the flock and its products [i.e., prohibition of trade with wool, raw milk (products), or live animals]. Moreover, **loss of income and threatened jobs** also must be considered. Although Q fever is listed by the OIE, it is not included within “Recommendations applicable to OIE Listed diseases and other diseases of importance to international trade” (5, 36). Since we recommend stopping animal trade from a positive herd, this could result in loss of income for the animal owners. This calculation and summary of financial costs shows that it is not feasible to implement this concept of a best practice MOSS.

Finally, the following steps are necessary to reduce costs in order to make this MOSS feasible. **Preventive measures** should replace testing wherever justifiable. Therefore, the focus on **nationwide vaccination** would be most useful, as this would guarantee lower pathogen shedding by infected small ruminants. Assuming the vaccination costs as stated above for all reproductive females (*n* = 1,270,011) in the German small ruminant population, a nationwide vaccination of these would include approximately 7,722,000 euros per year. If sample collection and testing are omitted completely, this would result in a cost reduction of 18,368,430 euros per year compared to the best practice concept [see Supplementary Material; (52, 53)]. Moreover, current studies are investigating if the **vaccination dose for sheep can be reduced by half** (1 ml per dose) compared to goats (25). Other studies are looking at whether **exclusive vaccination of gimmers** would be sufficient to prevent a positive flock status (68). These approaches would also further reduce costs. Unfortunately, it is not possible to avoid **sample collection and testing in cases of close contact** between small ruminants and the public because vaccination does not prevent pathogen shedding completely (5). On the other hand, vaccination can greatly reduce the risk of Q fever infection in the human population. For further cost reductions, it must also be considered that testing such a large number of animals means that **costs for sample testing and vaccination can be negotiated** between the national veterinary health authority and the industry, resulting in lower costs for each district as in our calculation above. In addition, costs could be saved if the **sample collection** was carried out by independent persons, such as employees of the responsible chamber of agriculture or Animal Disease Founds, who have lower labor costs as veterinarians. However, it must be ensured that employees have the necessary knowledge to guarantee the quality of the samples. Furthermore, it could also be considered whether the animal owners themselves could collect the samples. However, this raises several problems. First, it would have to be ensured that all animal owners have the necessary knowledge to carry out the sampling correctly. Second, the animal owners are directly affected by the results of the samples, which could make them prejudiced in taking the samples.

Regarding MOSS coordination, sample collection and testing as well as vaccination costs, it has to be decided which stakeholder group should participate in the financing. **Animal owners** could be a possible group as it is their responsibility not to compromise public health by animal husbandry (69). On the other hand, animal owners cannot be expected to solely take over these high costs and the responsibility. Therefore, the costs should be shared or assumed. While goat owners may be more willing to accept financial costs, as Q fever leads to losses for their animals (i.e., higher abortion rate), in contrast, sheep owners might be less willing to pay as Q fever usually does not show any health problems in sheep. Given the previous approaches in Germany, it is most likely that financial coverage would be taken over by the **Animal Disease Funds**. In addition, the public health authorities and veterinary health authorities should be considered in regard to joint (cost) management. This is because measures can only be ordered by the public health authorities. This is based on the IfSG. However, veterinary expertise is needed for disease control measures in small ruminant flocks, too. Therefore, it is desirable that an interdisciplinary team coordinates measures. In addition to the legal basis of the IfSG, both public and veterinary health authorities are responsible for the maintenance of public health as a common good. Although infection with *C. burnetii* poses a greater risk to human health than to animal health, joint financing by animal owners, Animal Disease Founds as well as the public health authorities and the veterinary health authorities is a logical conclusion.

Financial benefits arise from the prevention of human Q fever cases. Therefore, each case that can be prevented by this MOSS saves costs for **physicians, laboratories, medicine, hospitalization, and nonproductive time of humans**. “Expressed in disease adjusted life years (DALYs), Q fever ranked 12th of 32 infectious diseases in the Netherlands over the period 2007–2011, using the methodology developed under the Burden of Communicable Diseases in Europe (BCoDE) project. … The healthcare-associated costs of the Q fever epidemic in the Netherlands was estimated at €18.4–26.5 million and the productivity loss at an additional €1.3–10.3 million” (70). Hence, though not precisely estimable, costs can be high, and outbreaks may affect the health system considerably. Therefore, every preventive measure is financially preferable to ongoing passive monitoring as it is currently in place. The financial benefit to small ruminant owners and the associated incentive to participate is difficult to recognize at the beginning of MOSS implementation. Animal owners are afraid of the financial damage that a positive test will bring. At this point, however, it is very important to emphasize that an active MOSS also leads to **quality awareness**, which in turn brings financial benefits. Once MOSS is implemented nationwide and an awareness of this zoonosis among small ruminant owners, public, veterinary, and human health professionals is available, small ruminant owners can use a negative flock status to prove the quality of their action measures against Q fever. As a financial benefit, animal owners of flocks with a negative test result could sell raw milk (products) for a better price (e.g., by self-marketing or selling to dairies) and escape restrictions such as migrating ban or culling of animals. This MOSS can therefore be the basis for creating a quality mark “Q fever free” for small ruminant livestock in the future. In addition, this active MOSS ensures that Q fever in small ruminant livestock is controlled nationwide and thus also reduces the **risk of pathogen introduction** into Q fever free flocks, e.g., by additional purchases. Therefore, a financial benefit is that Q fever in small ruminant livestock will be less widespread after MOSS implementation and thus fewer costs for **action measures** will be necessary in future.

Emotional costs include **stigmatism** of people in the small ruminant sector, **existential fear** of animal owners and **anxiety of the public about infection**. These costs cannot be enumerated pecuniary, but they have to be considered during the implementation process and when communicating with stakeholders. This MOSS can only act with high efficiency if emotions of animal owners and the public are taken seriously and are addressed properly.

Finally, emotional benefits evolve from protection of the public against Q fever while cooperating with animal owners. Knowing about positive flock status enables animal owners to take safety measures and thus demonstrate their **sense of responsibility** toward their fellow humans. Negative flock status can gain the **confidence** of the public in the animal owners and lead to economic strengthening of their business. Small ruminants are a considerable component of German livestock production especially for landscape conservation and are popular in the private sector. This MOSS does not want to impair the husbandry of small ruminants in Germany. Rather, it intends to support the small ruminant sector with regard to their responsibility in **zoonosis prevention**. Therefore, an emotional benefit is that stakeholders will work together in reaching this aim in a One **Health approach**.

## Conclusion

In conclusion, this concept of active surveillance of Q fever in small ruminant livestock focuses on an early warning system in order to prevent Q fever infections in the human population. Considering a best practice approach, the aim is to identify flocks currently shedding the pathogen. Flocks should be considered positive if at least one pool of vaginal swabs is positive by PCR. The surveillance approach culminates in flock-specific action and control measures for the affected flocks. If this best practice concept were to be implemented, a huge conflict between economic costs and feasibility would emerge. Therefore, to maintain the aim of this MOSS (prevention of human cases by detecting small ruminant shedders), modifications of the concept are necessary. The system has been developed to serve as a basis for the introduction of a nationwide mandatory surveillance system in the future. Even without the context of subsequent obligation to participate or implement control measures, it is always a challenge to balance necessities and practicability when developing a MOSS.

Currently, successful implementation of this early warning system depends on **voluntary** participation of animal owners. For nationwide and mandatory implementation of this MOSS, a revision of the German law is necessary. Only if the active MOSS is ordered by law can Q fever be prevented safely among the public. In the meantime, and after legal obligation, the most important prerequisite for successful prevention of pathogen transmission is close cooperation between public health authorities and veterinary health authorities at the national and local levels as well as willingness to learn about the possibilities and challenges of the other parties. Furthermore, good and trusting communication with other stakeholders, especially with the animal owners, is mandatory. After successful implementation of this concept focusing on Q fever in small ruminant livestock, a further monitoring and surveillance system for Q fever in other domestic and wild mammals as a target group should be considered. This further development could provide insights into a possible pathogen reservoir in Germany and expand health protection for the population.

In conclusion, this surveillance system is built at the interface of animal and public health, thereby acting as a veterinary public health tool. The responsibility of veterinary and human medicine for public health is already well-recognized, and the One Health concept should be put into practice with the early warning system for Q fever presented here.

## Data Availability Statement

The original contributions presented in the study are included in the article/Supplementary Material, further inquiries can be directed to the corresponding author/s.

## Author Contributions

All authors contributed to the article and approved the submitted version.

## Conflict of Interest

The authors declare that the research was conducted in the absence of any commercial or financial relationships that could be construed as a potential conflict of interest.

## References

[1] Rodolakis A Q fever, state of art: epidemiology, diagnosis and prophylaxis. Small Rumin Res. (2006) 62:121–4. 10.1016/j.smallrumres.2005.07.038

[2] Bundesinstitut für Risikobewertung (BfR) Stellungnahmen 2010 Q-Fieber: Übertragung von Coxiella burnetii Durch den Verzehr von Lebensmitteln Tierischer Herkunft Unwahrscheinlich, Stellungnahme Nr. 018/2010 des BfR vom 15. Available online at: https://www.bfr.bund.de/de/bfr_stellungnahmen_2010.html (accessed April 02, 2020).

[3] Jones RM Nicas M Hubbard AE Reingold AL The infectious dose of *Coxiella burnetii* (q fever). Appl Biosaf. (2006) 11:32–41. 10.1177/153567600601100106

[4] Todkill D Fowler T Hawker JI. Estimating the incubation period of acute q fever, a systematic review. Epidemiol Infect. (2018) 146:665–72. 10.1017/S095026881700303X 29559012 PMC9134354

[5] World Organisation for Animal Health (OIE) Manual of Diagnostic Tests and Vaccines for Terrestrial Animals 2019 Chapter 3.1.16 Q Fever (NB: Version adopted in May 2018) Available online at: https://www.oie.int/standard-setting/terrestrial-manual/access-online/ (accessed March 11, 2020).

[6] Tissot-Dupont H Raoult D Clinical aspects, diagnosis, and treatment of Q fever. In: Raoult D Parola P editors. Rickettsial Diseases. Boca Raton, FL: CRC Press (2007). p. 291–9. 10.3109/9781420019971.021

[7] Angelakis E Raoult D Q fever. Vet Microbiol. (2010) 140:297–309. 10.1016/j.vetmic.2009.07.016 19875249

[8] Morroy G Keijmel SP Delsing CE Bleijenberg G Langendam M Timen A. Fatigue following acute q-fever: a systematic literature review. PLoS ONE. (2016) 11:e155884. 10.1371/journal.pone.0155884 27223465 PMC4880326

[9] Robert Koch-Institut (RKI) RKI-ratgeber infektionskrankheiten - merkblätter für ärzte, Q-fieber. Epidemiol Bull. (2002) 37:313–6.

[10] González-Barrio D Ruiz-Fons F. *Coxiella burnetii* in wild mammals: a systematic review. Transbound Emerg Dis. (2019). 66:662–71. 10.1111/tbed.13085 30506629

[11] Georgiev M Afonso A Neubauer H Needham H Thiéry R Rodolakis A. Q fever in humans and farm animals in four European countries, 1982 to 2010. Euro Surveill. (2013) 18:13–25. 23449232

[12] Plummer PJ McClure JT Menzies P Morley PS Van den Brom R Van Metre DC. Management of *Coxiella burnetii* infection in livestock populations and the associated zoonotic risk: a consensus statement. J Vet Intern Med. (2018) 32:1481–94. 10.1111/jvim.15229 30084178 PMC6189356

[13] Ruiz-Fons F González-Barrio D Aguilar-Ríos F Soler AJ Garde JJ Gortázar C. Infectious pathogens potentially transmitted by semen of the black variety of the manchega sheep breed: health constraints for conservation purposes. Anim Reprod Sci. (2014) 149:152–7. 10.1016/j.anireprosci.2014.07.006 25066603

[14] Wolf A Prüfer TL Schoneberg C Campe A Runge M Ganter M. Prevalence of *Coxiella burnetii* in German sheep flocks and evaluation of a novel approach to detect an infection via preputial swabs at herd-level. Epidemiol Infect. (2020) 148:e88. 10.1017/S0950268820000801 32172709 PMC7118722

[15] Körner S Makert GR Mertens-Scholz K Henning K Pfeffer M Starke A. Uptake and fecal excretion of *Coxiella burnetii* by *Ixodes ricinus* and *Dermacentor marginatus* ticks. Paras Vectors. (2020) 13:1–11. 10.1186/s13071-020-3956-z 32059686 PMC7023696

[16] Nusinovici S Hoch T Brahim ML Joly A Beaudeau F. The effect of wind on *Coxiella burnetii* transmission between cattle herds: a mechanistic approach. Transbound Emerg Dis. (2017) 64:585–92. 10.1111/tbed.12423 26392118

[17] Rodolakis A Q fever in dairy animals. Ann NY Acad Sci. (2009) 1166:90–3. 10.1111/j.1749-6632.2009.04511.x 19538267

[18] Berri M Souriau A Crosby M Crochet D Lechopier P Rodolakis A. Relationships between the shedding of *Coxiella burnetii*, clinical signs and serological responses of 34 sheep. Vet Rec. (2001) 148:502–5. 10.1136/vr.148.16.502 11345992

[19] Guatteo R Joly A Beaudeau F. Shedding and serological patterns of dairy cows following abortions associated with *Coxiella burnetii* DNA detection. Vet Microbiol. (2012) 155:430–3. 10.1016/j.vetmic.2011.09.026 21996545

[20] Agerholm JS. *Coxiella burnetii* associated reproductive disorders in domestic animals-a critical review. Acta Vet Scand. (2013) 55:13. 10.1186/1751-0147-55-13 23419216 PMC3577508

[21] Bouvery N Souriau A Lechopier P Rodolakis A. Experimental *Coxiella burnetii* infection in pregnant goats: excretion routes. Vet Res BioMed Centr. (2003) 34:423–33. 10.1051/vetres:2003017 12911859

[22] Berri M Rousset E Champion JL Russo P Rodolakis A. Goats may experience reproductive failures and shed *Coxiella burnetii* at two successive parturitions after a q fever infection. Res Vet Sci. (2007) 83:47–52. 10.1016/j.rvsc.2006.11.001 17187835

[23] Palmer NC Kierstead M Key DW Williams JC Peaceock MG Vellend H. Placentitis and abortion in goats and sheep in ontario by *Coxiella burnetii*. Can Vet J. (1983) 24:60–1. 17422227 PMC1790252

[24] European Centre for Disease Prevention and Control Q fever. In: ECDC. Annual Epidemiological Report for 2018. Stockholm: ECDC (2019).

[25] Bauer BU Runge M Campe A Henning K Mertens-Scholz K Boden K Coxiella burnetii: A Review Focusing on Infections in German Sheep and Goat Flocks. Berl Münch Tierärztl Wochenschr 2020. Available online at: https://vetline.de/icoxiella-burnetiii-ein-uebersichtsartikel-mit-fokus-auf-das-infektionsgeschehen-in-deutschen-schaf-und-ziegenherden/150/3216/111683 (accessed April 15, 2020).

[26] Hellenbrand W Schöneberg I Pfaff G Kramer M Steng G Reintjes R The relevance of *Coxiella burnetii* infections in animals for q fever in humans - measures for prevention and control. Tierärztl Prax. (2005) 33:5–11. 10.1055/s-0038-1624108

[27] Hellenbrand W Breuer T Petersen L. Changing epidemiology of q fever in Germany, 1947-1999. Emerg Infect Dis. (2001) 7:789–96. 10.3201/eid0705.010504 11747689 PMC2631891

[28] Robert Koch-Institut (RKI) Infektions Epidemiologisches Jahrbuch Meldepflichtiger Krankheiten für Das Jahr 2018. Berlin: Robert Koch-Institut (2019).

[29] Porten K Rissland J Tigges A Broll S Hopp W Lunemann M. A super-spreading ewe infects hundreds with q fever at a farmers' market in Germany. BMC Infect Dis. (2006) 6:147. 10.1186/1471-2334-6-147 17026751 PMC1618839

[30] Federal Institute for Consumer Health Protection and Veterinary Medicine (BgVV) Bericht über die epidemiologische Situation der Zoonosen in Deutschland für 1998, Übersicht über die Meldungen der Bundesländer. In: Hartung M editor. BgVV-Hefte XX/1999. Berlin: Federal Institute for Consumer Health Protection and Veterinary Medicine (BgVV) (1999). p. 172.

[31] Clark NJ Magalhaes RJS. Airborne geographical dispersal of q fever from livestock holdings to human communities: a systematic review and critical appraisal of evidence. BMC Infect Dis. (2018) 18:218. 10.1186/s12879-018-3135-4 29764368 PMC5952368

[32] Sidi-Boumedine K Rousset E Henning K Ziller M Niemczuck K Roest HIJ Development of harmonised schemes for the monitoring and reporting of q-fever in animals in the European Union. EFSA Support Publ. (2010) 7:1–48. 10.2903/sp.efsa.2010.EN-48

[33] Ganter M Zoonotic risks from small ruminants. Vet Microbiol. (2015) 181:53–65. 10.1016/j.vetmic.2015.07.015 26275853

[34] Bond KA Vincent G Wilks CR Franklin L Sutton B Stenos J. One health approach to controlling a q fever outbreak on an Australian goat farm. Epidemiol Infect. (2016) 144:1129–41. 10.1017/S0950268815002368 26493615 PMC4825098

[35] George M Reich A Cussler K Jehl H Burckhardt F. Live cell therapy as potential risk factor for q fever. Emerg Infect Dis. (2017) 23:1210–2. 10.3201/eid2307.161693 28296631 PMC5512499

[36] World Organisation for Animal Health (OIE) Animal Terrestrial Health Code. (2019). Available online at: https://www.oie.int/index.php?id=169&L=0&htmfile=sommaire.htm (accessed March 11, 2020).

[37] Infektionsschutzgesetz vom 20 Juli 2000 (BGBl. I S. 1045), Das Zuletzt Durch Artikel 3 des Gesetzes Vom 27. März 2020 (BGBl. I S. 587) Geändert Worden IST. Available online at: https://www.gesetze-im-internet.de/ifsg/BJNR104510000.html (accessed March 15, 2020).

[38] Tiergesundheitsgesetz in der Fassung der Bekanntmachung vom 21 November 2018 (BGBl. I S. 1938), Das Zuletzt Durch Artikel 100 des Gesetzes vom 20. November 2019 (BGBl. I S. 1626) Geändert Worden IST. Available online at: https://www.gesetze-im-internet.de/tiergesg/BJNR132400013.html (accessed March 15, 2020).

[39] Verordnung über meldepflichtige Tierkrankheiten in der Fassung der Bekanntmachung vom 11 Februar 2011 (BGBl. I S. 252), Die Zuletzt Durch Artikel 381 der Verordnung vom 31. August 2015 (BGBl. I S. 1474) Geändert Worden IST. Available online at: https://www.gesetze-im-internet.de/tkrmeldpflv_1983/BJNR010950983.html (accessed March 15, 2020).

[40] Chemisches und Veterinäruntersuchungsamt (CVUA) Stuttgart Leitfaden zum Q-Fieber Baden-Württemberg: Empfehlungen zur Bekämpfung des Q-Fiebers bei kleinen Wiederkäuern in Baden-Württemberg. Stuttgart (2017).

[41] Federal Ministry of Food and Agriculture (BMEL) Bekanntmachung von Empfehlungen für Hygienische Anforderungen an das Halten von Wiederkäuern vom 7 Juli 2014 (BAnz. AT 01.08.2014 BI) Geändert Durch Erste Bekanntmachung der Änderung der Bekanntmachung von Empfehlungen für Hygienische Anforderungen an das Halten von Wiederkäuern vom 19. August 2014 (BAnz AT 28.08.2014 B1). Available online at: https://www.bmel.de/DE/Tier/Tiergesundheit/_texte/EmpfehlungenHygiene.html (accessed March 15, 2020).

[42] Salman MD Animal Disease Surveillance and Survey Systems: Methods and Applications. Iowa, IA: Iowa State Press (2003). p. 1–222. 10.1002/9780470344866

[43] Doherr MG Audige L. Monitoring and surveillance for rare health-related events: a review from the veterinary perspective. Philos Trans R Soc Lond B. (2001) 356:1097–106. 10.1098/rstb.2001.0898 11516387 PMC1088504

[44] Hilbert A Schmoock G Lenzko H Moog U Diller R Fröhlich A. Prevalence of *Coxiella burnetii* in clinically healthy German sheep flocks. BMC Res Notes. (2012) 5:152. 10.1186/1756-0500-5-152 22429653 PMC3351016

[45] Roest HIJ Tilburg JJHC Van der Hoek W Vellema P Van Zijderveld FG Klaassen CHW. Review article, the q fever epidemic in The Netherlands: history, onset, response and reflection. Epidemiol Infect. (2011) 139:1–12. 10.1017/S0950268810002268 20920383

[46] Hoinville LJ Animal health surveillance terminology, final report from Pre-ICAHS Workshop, July 2013 (version 1.2). In: International Conference on Animal Health Surveillance (ICAHS). La Havana: CUB (2013).

[47] Hoinville LJ Alban L Drewe JA Gibbens JC Gustafson L Häsler B. Proposed terms and concepts for describing and evaluating animal-health surveillance systems. Prev Vet Med. (2013) 112:1–12. 10.1016/j.prevetmed.2013.06.006 23906392

[48] Comin A Haesler B Hoinville LJ Peyre M Dórea F Schauer B RISKSUR tools: taking animal health surveillance into the future through interdisciplinary integration of scientific evidence. In: Annual Meeting of the Society for Veterinary Epidemiology and Preventive Medicine. Elsinore: DNK (2016).

[49] Sargeant JM O'Connor AM. Issues of reporting in observational studies in veterinary medicine. Prev Vet Med. (2014). 113:323–30. 10.1016/j.prevetmed.2013.09.004 24139690

[50] Runge M Binder A Schotte U Ganter M Investigations concerning the prevalenceof *Coxiella burnetii* and *Clamydia abortus* in sheep in correlation with management systems and abortion rate in Lower saxony in 2004. Berl Munch Tierarztl Wochenschr. (2012) 125:10–5. 10.2376/0005-9366-125-138 22515032

[51] Dohoo IR Martin W Stryhn H Veterinary Epidemiologic Research. Charlottetown, PE: VER Inc. (2010). p. 1–865.

[52] Federal Statistical Office of Germany (Destatis) Ergebnis - 41141-0019 Landwirtschaftliche Betriebe mit Viehhaltung, Viehbestand: Bundesländer, Stichtag, Tierarten (01.03.2016). Available online at: https://www-genesis.destatis.de/genesis/online/data;sid=BD01EDE95F2DAE53798F8BBFCB748D7D.GO_2_1?operation=abruftabelleBearbeiten&levelindex=1&levelid=1568200076966&auswahloperation=abruftabelleAuspraegungAuswaehlen&auswahlverzeichnis=ordnungsstruktur&auswahlziel=werteabruf&selectionname=41141-0019&auswahltext=&werteabruf=Werteabruf (accessed September 11, 2019).

[53] Federal Statistical Office of Germany (Destatis) Federal Ministry of Food and Agriculture (BMEL) Statistik und Berichte des BMEL, Landwirtschaft, Tierhaltung, Schafe, MBT-0117490-0000 Betriebe mit Schafhaltung nach Bestandsgrößenklassen. Available online at: https://www.bmel-statistik.de/landwirtschaft/tierhaltung/schafhaltung/ (accessed March 24, 2020).

[54] Debeljak Z Medic S Baralic M Andric A Tomic A Vidanovic D. Clinical, epidemiological and epizootic features of a Q fever outbreak in the border region between Serbia and Montenegro. J Infect Dev Ctries. (2018) 12:290–6. 10.3855/jidc.9918 31865289

[55] Anderson AD Szymanski TJ Emery MP Kohrs PH Bjork AC Marsden-Haug N. Epizootiological investigation of a q fever outbreak and implications for future control strategies. JAVMA. (2015) 247:1379–86. 10.2460/javma.247.12.1379 26642131

[56] Bauer B Prüfer L Walter M Ganter I Frangoulidis D Runge M. Comparison of *Coxiella burnetii* excretion between sheep and goats naturally infected with one cattle-associated genotype. Pathogens. (2020) 9:15. 10.3390/pathogens9080652 32823701 PMC7459479

[57] Roest HJ van Gelderen B Dinkla A Frangoulidis D van Zijderveld F Rebel J. Q Fever in pregnant goats: pathogenesis and excretion of *Coxiella burnetii*. PLoS ONE. (2012). 7:e48949. 10.1371/journal.pone.0048949 23152826 PMC3494687

[58] Friedrich-Loeffler-Institut (FLI) Amtliche Methodensammlung Q-Fieber. Greifswald-Insel Riems (2018).

[59] Rousset E Berri M Durand B Dufour P Prigent M Delcroix T. *Coxiella burnetii* shedding routes and antibody response after outbreaks of q fever-induced abortion in dairy goat herds. Appl Environ Microbiol. (2009) 75:428–33. 10.1128/AEM.00690-08 19011054 PMC2620711

[60] Kreienbrock L Pigeot I Ahrens W Epidemiologische Methoden. Berlin; Heidelberg: Springer (2012). p. 492 10.1007/978-3-8274-2334-4

[61] Arricau-Bouvery N Souriau A Bodier C Dufour P Rousset E Rodolakis A. Effect of vaccination with phase I and phase II *Coxiella burnetii* vaccines in pregnant goats. Vaccine. (2005) 23:4392–402. 10.1016/j.vaccine.2005.04.010 16005747

[62] Rahaman MR Milazzo A Marshall H Bi P. Is a one health approach utilized for q fever control? A comprehensive literature review. Int J Environ Res Public Health. (2019) 16:730. 10.3390/ijerph16050730 30823481 PMC6427780

[63] de Vries DH Kinsman J Takacs J Tsolova S Ciotti M. Methodology for assessment of public health emergency preparedness and response synergies between institutional authorities and communities. BMC Health Serv Res. (2020) 20:411. 10.1186/s12913-020-05298-z 32393259 PMC7212582

[64] Greiner M Baumann M Campe A Doherr MG Gareis M Greif G Zur rolle der veterinärmedizin im bereich public health. Deutsch Tierärzteblatt. (2017) 65:158–61.

[65] Tierärztegebührenordnung vom 28 Juli 1999 (BGBl. I S. 1691), die Zuletzt Durch Artikel 1 der Verordnung vom 10. Februar 2020 (BGBl. I S. 158) Geändert Worden IST. Available online at: https://www.gesetze-im-internet.de/got/BJNR169100999.html (accessed March 15, 2020).

[66] Gebührenordnung für die Verwaltung im Bereich des Verbraucherschutzes und des Veterinärwesens (GOVV) vom 29 November 2014, Letzte Berücksichtigte Änderung: Anlage Geändert Durch Verordnung vom 16.01.2020 (Nds. GVBl. S. 4). Available online at: http://www.nds-voris.de/jportal/portal/t/1bqx/page/bsvorisprod.psml?pid=Dokumentanzeige&showdoccase=1&js_peid=Trefferliste&documentnumber=1&numberofresults=1&fromdoctodoc=yes&doc.id=jlr-VetVwGOND2014V11Anlage&doc.part=X&doc.price=0.0#focuspoint (accessed March 15, 2020).

[67] Ständige Impfkommission Veterinärmedizin (StIKo Vet) am Friedrich-Loeffler-Institut (FLI) Leitlinie zur Impfung von Rindern und kleinen Wiederkäuern. Greifswald-Insel Riems (2018).

[68] Bauer BU Janowetz B Turowski V Ambros C Alex M Domes U Verlauf der Coxiellose in Einer Schafherde Nach Jährlicher Grundimmunisierung der Zutreter [Internet]. Tagung der DVG-Fachgruppe Krankheiten kleiner Wiederkäuer: Greifswald - Insel Riems (2019). p. 83–5. Available online at: https://elib.tiho-hannover.de/receive/tiho_mods_00003290

[69] Coors W Das Tierseuchenentschädigungsrecht. Hamburg: Jur. Fakultät Universität Hamburg (1966).

[70] Discontool- Research gaps for improving infectious disease control in animals Diseases - Q-Fever. Available online at: https://discontools.eu/database/57-q-fever.html (accessed February 27, 2020).

